# Adaptation in a Fibronectin Binding Autolysin of *Staphylococcus saprophyticus*

**DOI:** 10.1128/mSphere.00511-17

**Published:** 2017-11-29

**Authors:** Tatum D. Mortimer, Douglas S. Annis, Mary B. O’Neill, Lindsey L. Bohr, Tracy M. Smith, Hendrik N. Poinar, Deane F. Mosher, Caitlin S. Pepperell

**Affiliations:** a Department of Medical Microbiology and Immunology, School of Medicine and Public Health, University of Wisconsin—Madison, Madison, Wisconsin, USA; b Microbiology Doctoral Training Program, University of Wisconsin—Madison, Madison, Wisconsin, USA; c Department of Biomolecular Chemistry, School of Medicine and Public Health, University of Wisconsin—Madison, Madison, Wisconsin, USA; d Laboratory of Genetics, University of Wisconsin—Madison, Madison, Wisconsin, USA; e Department of Medicine, Division of Infectious Diseases, School of Medicine and Public Health, University of Wisconsin—Madison, Madison, Wisconsin, USA; f McMaster Ancient DNA Centre, Department of Anthropology, McMaster University, Hamilton, Ontario, Canada; g Department of Biology, McMaster University, Hamilton, Ontario, Canada; h Michael G. DeGroote Institute for Infectious Disease Research, McMaster University, Hamilton, Ontario, Canada; i Humans and the Microbiome Program, Canadian Institute for Advanced Research, Toronto, Ontario, Canada; Antimicrobial Development Specialists, LLC

**Keywords:** *Staphylococcus saprophyticus*, adhesins, evolution, positive selection, urinary tract infection

## Abstract

Human-pathogenic bacteria are found in a variety of niches, including free-living, zoonotic, and microbiome environments. Identifying bacterial adaptations that enable invasive disease is an important means of gaining insight into the molecular basis of pathogenesis and understanding pathogen emergence. *Staphylococcus saprophyticus*, a leading cause of urinary tract infections, can be found in the environment, food, animals, and the human microbiome. We identified a selective sweep in the gene encoding the Aas adhesin, a key virulence factor that binds host fibronectin. We hypothesize that the mutation under selection (*aas*_2206A>C) facilitates colonization of the urinary tract, an environment where bacteria are subject to strong shearing forces. The mutation appears to have enabled emergence and expansion of a human-pathogenic lineage of *S. saprophyticus*. These results demonstrate the power of evolutionary genomic approaches in discovering the genetic basis of virulence and emphasize the pleiotropy and adaptability of bacteria occupying diverse niches.

**IMPORTANCE** *Staphylococcus saprophyticus* is an important cause of urinary tract infections (UTI) in women; such UTI are common, can be severe, and are associated with significant impacts to public health. In addition to being a cause of human UTI, *S. saprophyticus* can be found in the environment, in food, and associated with animals. After discovering that UTI strains of *S. saprophyticus* are for the most part closely related to each other, we sought to determine whether these strains are specially adapted to cause disease in humans. We found evidence suggesting that a mutation in the gene *aas* is advantageous in the context of human infection. We hypothesize that the mutation allows *S. saprophyticus* to survive better in the human urinary tract. These results show how bacteria found in the environment can evolve to cause disease.

## Abstract (precis)

*Staphylococcus saprophyticus* is an important cause of urinary tract infections (UTI) in women; such UTI are common, can be severe, and are associated with significant impacts to public health. In addition to being a cause of human UTI, *S. saprophyticus* can be found in the environment, in food, and associated with animals. After discovering that UTI strains of *S. saprophyticus* are for the most part closely related to each other, we sought to determine whether these strains are specially adapted to cause disease in humans. We found evidence suggesting that a mutation in the gene *aas* is advantageous in the context of human infection. We hypothesize that the mutation allows *S. saprophyticus* to survive better in the human urinary tract. These results show how bacteria found in the environment can evolve to cause disease.

## INTRODUCTION

Urinary tract infections (UTI) are a global health problem of major significance, with an estimated annual incidence of 150 to 250 million and a lifetime risk of 50% among women (1–3). The associated costs for individuals and health care systems are substantial, with recent estimates from the United States numbering in the billions of dollars per year (4). UTI are also associated with severe complications such as pyelonephritis, sepsis, and premature labor (4). *Staphylococcus saprophyticus* is second only to *Escherichia coli* as a cause of UTI in reproduction-aged women (5, 6).

*S. saprophyticus* can be found in diverse niches, including the environment, foods, and livestock, and as a pathogen and commensal of humans. Several features of the epidemiology of *S. saprophyticus* suggest that infections leading to UTI are acquired from the environment rather than as a result of person-to-person transmission (7). This implies that adoption of the pathogenic niche by *S. saprophyticus* has not entailed a tradeoff in its ability to live freely in the environment. A recent PCR-based survey of virulence factors in clinical and animal-associated isolates showed that *dsdA*, a gene encoding d-serine deaminase that is important for survival in urine (8), and *uafA* and *aas*, genes encoding adhesins that mediate binding to uroepithelium (9, 10), were present in all isolates surveyed (11), suggesting an underlying pleiotropy, with these virulence factors playing important roles in the diverse environments occupied by *S. saprophyticus*.

The human urinary tract could represent an evolutionary dead end for *S. saprophyticus*, with “virulence factors” such as DsdA, UafA, and Aas serving an essential function in the primary environmental niche and enabling invasion of the urinary tract as an accidental by-product of this unknown primary function. In such a case, we would expect urinary isolates to be interspersed throughout a phylogeny of isolates from the primary niche(s). However, our previous research (7) indicated that human urinary tract infections are associated with a specific lineage of *S. saprophyticus*. Invasion of the human urinary tract enables *S. saprophyticus* to grow to high numbers in urine, isolated from competing bacterial species, before being redeposited in the environment. This is analogous to *Vibrio cholerae*, which cycles through human and environmental niches and grows to high abundance in the human gut before being deposited in the environment via stool (12, 13). Based on our previous observations and the example provided by other human pathogens that cycle through the environment, we hypothesized that the human urinary tract is an ecologically important niche for *S. saprophyticus* and sought to identify genetic signatures of adaptation to this niche.

The increased availability of sequencing data has enabled comparative genomic approaches that have led to identification of changes in gene content in association with pathogen emergence and shifts in host association. Several notable human pathogens, including *Mycobacterium tuberculosis*, *Yersinia pestis*, and *Francisella tularensis*, are the product of a single emergence characterized by gene loss and horizontal acquisition of virulence factors (14–16). Similarly, genomic analysis of *Enterococcus faecium* revealed gene gains and losses affecting metabolism and antibiotic resistance in the emergence of a hypermutable hospital-adapted clade that coincided with the profound shift in hospital ecology caused by the development of antibiotics (17). Gene gains via recombination have also allowed *Staphylococcus aureus* ST71 to emerge into a bovine-associated niche (18).

Using contemporary and ancient genomic data from strains of *S. saprophyticus*, we found previously that UTI-associated lineages of *S. saprophyticus* were not associated with specific gene gains or losses; the evolutionary genetic processes underlying the adoption by *S. saprophyticus* of the human-pathogenic niche are likely more subtle than those previously described for canonical pathogens (7). Here we have identified one of the mechanisms underlying the adaptation of *S. saprophyticus* to the uropathogenic niche: a selective sweep in the Aas adhesin, which is associated with an apparently large-scale expansion into the human-pathogenic niche. This is, to our knowledge, the first identification of a single nucleotide sweep in a bacterium.

## RESULTS

**FIG 1:**
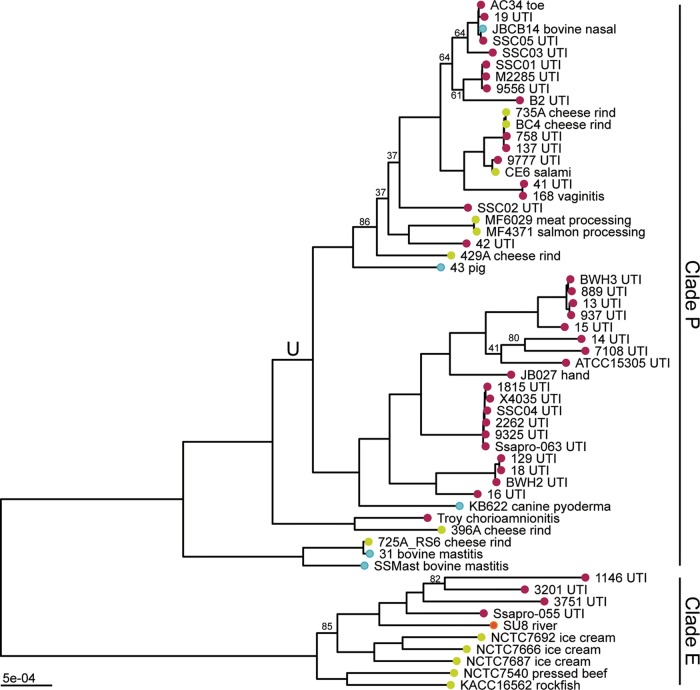
Maximum likelihood phylogeny of *S. saprophyticus*. Maximum likelihood phylogenetic analysis was performed in RAxML (92) using a whole-genome alignment with repetitive regions masked. The phylogeny is midpoint rooted, and nodes with bootstrap values of less than 90 are labeled. Branch lengths are scaled by the number of substitutions per site. Tips are colored based on the isolation source (pink, human; blue, animal; green, food; orange, environment). Tips are labeled with the isolate name and detailed source information. *S. saprophyticus* contains two major clades (clade P and clade E). Within clade P, there is a lineage enriched in human-pathogenic isolates (lineage U [branch labeled “U”]).

We reconstructed the phylogeny of *S. saprophyticus* isolates (see Table S1 in the supplemental material) from a whole-genome alignment using maximum likelihood inference implemented in RAxML (Fig. 1). The bacterial isolates are separated into two clades, which we previously named clades P and E (7). In both clades, human-associated lineages are nested among isolates from diverse sources, including food (cheese rind, ice cream, meat), indoor and outdoor environments, and animals. Interestingly, cheese rinds harbor diverse strains of *S. saprophyticus*, which cluster with both human- and animal-pathogenic strains.

10.1128/mSphere.00511-17.1 TABLE S1  Accession numbers for *S. saprophyticus* isolates. Download TABLE S1, TXT file, 0.001 MB. Copyright © 2017 Mortimer et al. 2017 Mortimer et al. This content is distributed under the terms of the Creative Commons Attribution 4.0 International license.

Thirty-three of 37 modern, human-pathogenic isolates are found within a single lineage (that we term lineage U [for "UTI associated"]) with respect to which bovine-pathogenic (mastitis), food-associated isolates, and an ancient genome are basal. Given the association between this lineage and illness in humans, we were curious about its potential adaptation to the human-pathogenic niche. The placement of the 800-year-old strain between bovine-associated and human-associated lineages suggests that it could represent a generalist intermediate between human-adapted and bovid-adapted strains.

Core genome analysis of the 58 isolates of *S. saprophyticus* in our sample showed substantial variability in gene content; the core genome is composed of 1,798 genes, and there are an additional 7,110 genes in the pan-genome. We found previously that uropathogenic isolates of *S. saprophyticus* were not associated with any unique gene content (7). Given the variability in accessory gene content among the members of this larger sample of isolates, we decided to test for relative differences in accessory gene content between human clinical isolates and other isolates using Scoary (19), which performs a genome-wide association study (GWAS) using gene presence and absence. We did not identify any genes that were significantly associated with the human-pathogenic niche after correction for multiple-hypothesis testing using the Bonferroni method.

**FIG 2:**
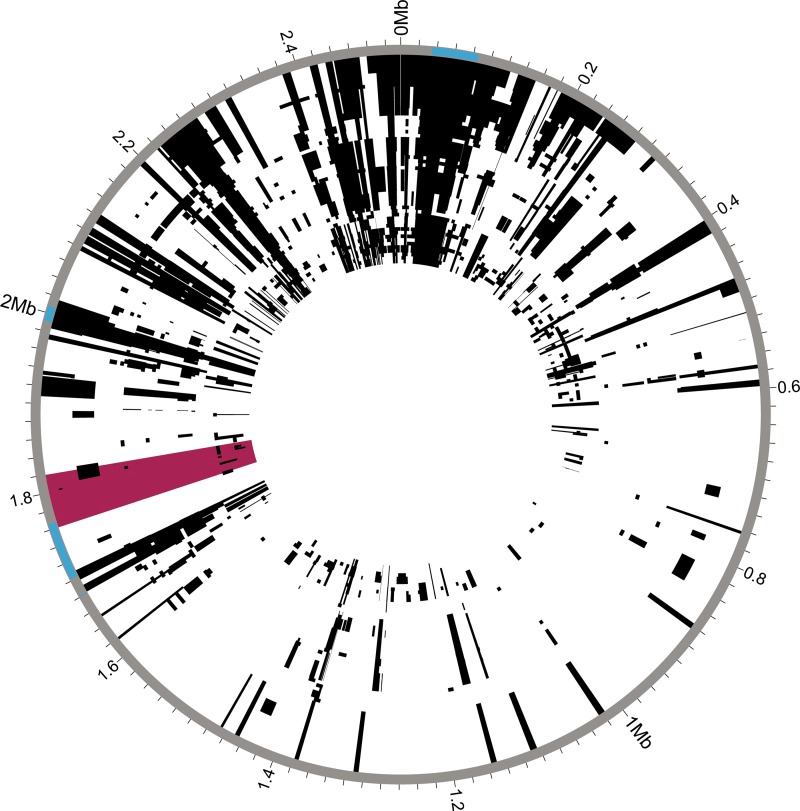
Recombination in *S. saprophyticus*. Recombinant regions in the whole-genome alignment of *S. saprophyticus* were identified using Gubbins (20). Mobile genetic elements are highlighted in blue on the outer rim. The window with low Tajima’s D and π values is highlighted in pink. Few recombination events are inferred within this region.

In addition to the observed gene content variability, analyses of the core genome also indicated relatively frequent recombination among *S. saprophyticus* isolates (Fig. 2). We identified recombinant regions with Gubbins (20), which identifies regions with high densities of substitutions. These results indicated that 70% of sites in the *S. saprophyticus* alignment had been affected by recombination. Recombination can affect bacterial evolution both by introducing novel polymorphisms from outside the population and by reshuffling alleles without increasing overall diversity. Considering sites that are reshuffled within the *S. saprophyticus* sample to be recombinant sites, we estimated a ratio of recombinant to nonrecombinant single nucleotide polymorphisms (SNPs) of 3.4. Considering only the SNPs that introduce novel diversity to be recombinant SNPs, our estimate of the ratio of recombinant SNPs to nonrecombinant SNPs is 0.51. The mean recombination-per-mutation (r/m) value for branches in the phylogeny is 0.82 (range, 0 to 7.6) as estimated by Gubbins. Removal of recombinant SNPs did not affect the topology of the maximum likelihood phylogeny. We observed regional patterns in the amount of recombination inferred, and, as expected, recombination appears to be frequent at mobile elements such as the staphylococcal cassette chromosomes (SCC_15305RM_ and SCC_15305cap_) and *v*Ss15305 (10).

**FIG 3:**
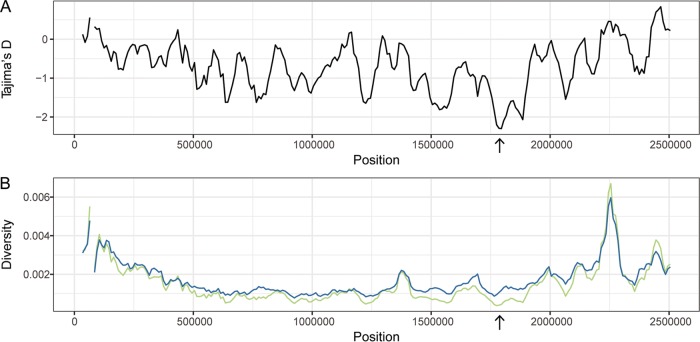
Sliding window analysis of diversity and neutrality statistics. Population genetic statistics were calculated for lineage U using EggLib (94). Windows were 50 kb in width with a step size of 10 kb. (A) Tajima’s D. (B) π (green) and θ (blue). The lowest values for Tajima’s D and π are found in the same window (1,760,000 to 1,820,000 bp; arrow).

Adaptation to a new environment may be facilitated by advantageous mutations that quickly rise in frequency, leaving a characteristic genomic imprint: reduced diversity at the target locus and nearby linked loci (i.e., selective sweep [21, 22]). In order for positive selection to be evident as a local reduction in diversity, there must be sufficient recombination for the target locus to be unlinked from the rest of the genome; for this reason, scans for sweeps have been used primarily for sexually reproducing organisms (23–26). As described above and in prior work (7), we found evidence of frequent recombination among *S. saprophyticus* isolates. We hypothesized that the transition of *S. saprophyticus* to the uropathogenic niche may have been driven by selection for one or more mutations that were advantageous in the new environment and that levels of recombination have been sufficient to preserve the signature of a selective sweep at loci under positive selection. We therefore used a sliding window analysis of diversity along the *S. saprophyticus* alignment as an initial screen for positive selection. We identified a marked regional decrease in nucleotide diversity (π) and in Tajima’s D (TD) that is specific to lineage U (Fig. 3); the TD values for this window were −0.38 and 0.94 for non-lineage U clade P isolates and clade E isolates, respectively. The region with a decreased π/TD ratio corresponded to bp 1760000 to 1820000 in *S. saprophyticus* ATCC 15305 and had the lowest values of π and TD in the entire alignment. We investigated the sensitivity of our sliding window analyses to sampling by randomly subsampling lineage U isolates to the same size as clade E (*n* = 10); we found the results to be robust with respect to changes in sampling scheme and size.

To complement the sliding window analysis and pinpoint candidate variants under positive selection, we used an approach based on allele frequency differences between bacterial isolates from different niches. We calculated Weir and Cockerham’s *F*_*ST*_ (27) for single nucleotide polymorphisms (SNPs) in the *S. saprophyticus* genome using human association and nonhuman association to define populations. The region of low π/TD included three nonsynonymous variants in the top 0.05% of the *F*_*ST*_ values (Table 1). One of these variants was fixed among human-associated isolates in lineage U (position 1811777 in ATCC 15305; *F*_*ST*_ = 0.48) and distinct from the ancestral (anc) allele found in basal lineages of clade P, including the ancient strain of *S. saprophyticus* Troy. This suggests that the variant may have been important in adaptation to the human urinary tract. To assess the significance of the *F*_*ST*_ value for this variant, we performed permutation analyses by randomly assigning isolates as human associated, and we did not achieve *F*_*ST*_ values higher than 0.28 in 100 permutations.

**TABLE 1:** Single nucleotide polymorphisms with *F*_*ST*_ values in the top 0.05% between 1,760,000 and 1,820,000 bp in ATCC 15305

Position	Frequency in human- associated isolates	Frequency in non-human- associated isolates	*F*_*ST*_ value	Type
1772616	0.72	0.16	0.5	Nonsynonymous
1797190	0.9	0.37	0.48	Synonymous
1808274	0.8	0.21	0.52	Synonymous
1811585	0.72	0.16	0.5	Synonymous
1811777	0.9	0.37	0.48	Nonsynonymous
1813204	0.74	0.16	0.53	Synonymous
1816895	0.77	0.05	0.71	Nonsynonymous
1818150	0.77	0.16	0.56	Intergenic
1818151	0.77	0.16	0.56	Intergenic
1818156	0.77	0.16	0.56	Intergenic

**FIG 4:**
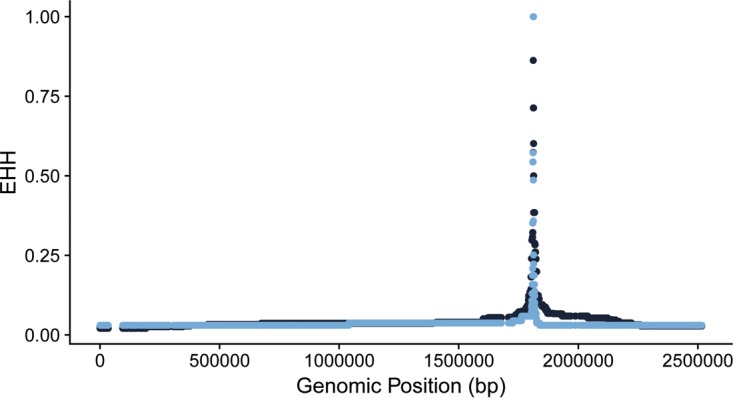
Extended haplotype homozygosity (EHH) of a single nucleotide polymorphism at position 1811777. EHH values for the ancestral allele are in light blue. EHH values for the derived allele are in dark blue.

Selective sweeps may be evident as a longer-than-expected haplotype block, since neutral variants linked to the adaptive mutation also sweep to high frequencies (28). Given the evidence suggesting that there was a selective sweep at this locus, we used haplotype-based statistics to test for such a signature in the *S. saprophyticus* alignment. Haplotype-based methods are hypothesized to not be applicable to bacteria due to differences between crossing-over and bacterial patterns of recombination (29), but the methods had not been tested in a scenario akin to a classical sweep, in which local changes in diversity and in the site frequency spectrum (SFS) have been observed. We found that the variant at position 181177 did show a signature of a sweep using the extended haplotype homozygosity (EHH) statistic (28) (Fig. 4). However, the variant did not have an extreme value of *nS*_L_, which compares haplotype homozygosity for ancestral and derived alleles (30), after normalization by the allele frequency.

**FIG 5:**
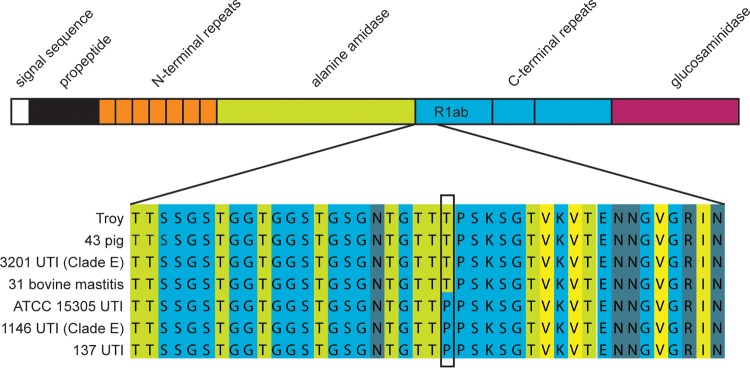
Nonsynonymous variant in Aas fibronectin binding repeat. (Top) Domains of Aas protein (adapted from reference 31. R1ab is the peptide used in the fibronectin and thrombospondin binding experiments. (Bottom) Alignment of a portion of R1 showing amino acid sequences in Aas from selected *S. saprophyticus* strains. Amino acids are colored based on their propensity to form beta strands (light green, high propensity; light blue, low propensity). The alignment visualization was created in JalView.

The variant of interest (*aas*_2206A>C) causes a threonine-to-proline change in the amino acid sequence of Aas, a bifunctional autolysin with a fibronectin binding domain (Fig. 5) (31). There are 8 additional nonsynonymous polymorphisms in the fibronectin binding domain; however, none are as highly associated with human-pathogenic isolates. Adhesins such as Aas are important in the pathogenesis of *S. saprophyticus* urinary tract infections, and the gene encoding Aas has been previously implicated as a virulence factor (9, 31, 32).

**FIG 6:**
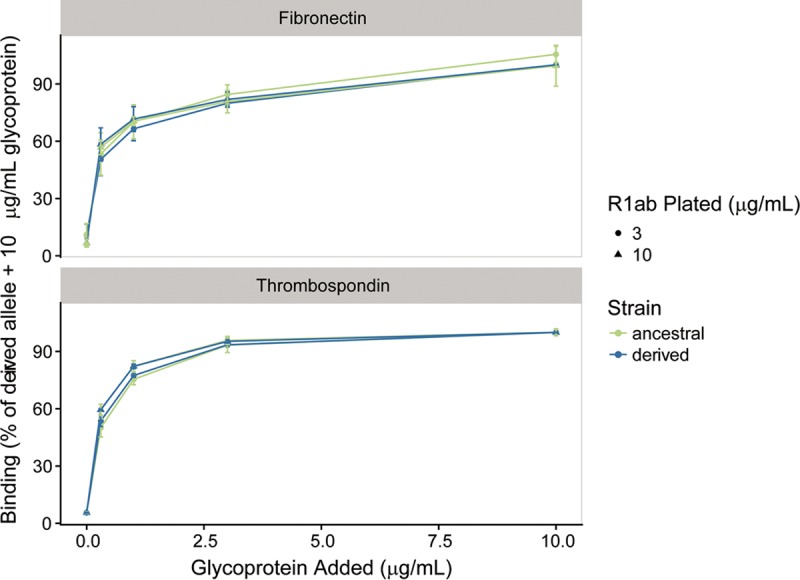
Fibronectin and thrombospondin binding to human-associated and ancestral strain Aas R1ab. ELISAs detecting the binding of soluble human fibronectin and thrombospondin to plates coated with Aas R1ab at 3 and 10 µg/ml were performed. Results have been normalized to the percentage of binding of 10 µg/ml glycoprotein to human-associated strain R1ab. Human and bovine fibronectin and human thrombospondin bound to the two constructs equally well.

The Aas variant is in a region known to bind fibronectin (Fig. 5) (31) and could be under selection because it affects adhesion to this host protein. We used enzyme-linked immunosorbent assays (ELISAs) to investigate potential effects of *aas*_2206A>C on binding to fibronectin and thrombospondin-1, which binds to this region of the homologous AtlE amidase from *Staphylococcus epidermidis* (33). Staphylococcal autolysins contains 3 C-terminal repeats (R1 to R3), which can each be divided into two subunits (a and b) based on structural information (34). We confirmed that Aas R1ab binds fibronectin and discovered that it also binds thrombospondin (Fig. 6); there was no detectable difference between the ancestral and derived R1ab alleles in binding to fibronectin or thrombospondin (human or bovine).

Interestingly, we observed several instances of recombination of the *aas* variant. In each case, the recombination event reinforced the association of the derived allele with human infection. Two of the non-human-associated bacterial isolates in lineage U—an isolate from a pig and a second from cheese rind—had evidence of a recombination event at the *aas* locus resulting in acquisition of the ancestral allele. Conversely, one of the human UTI isolates in clade E (for which the ancestral allele is otherwise fixed) acquired the derived *aas* variant.

**FIG 7:**
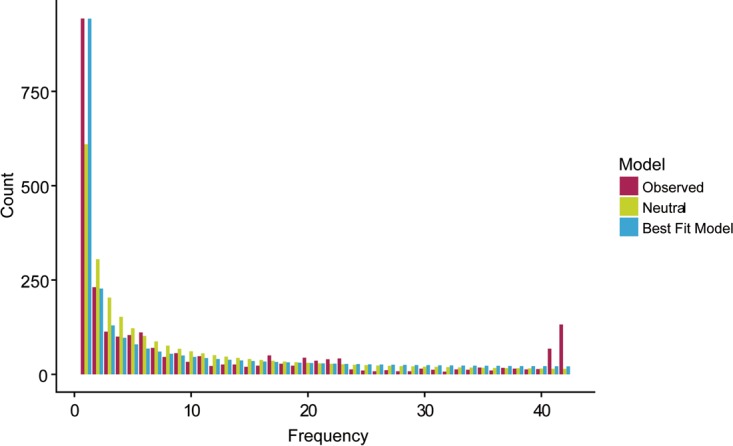
Site frequency spectrum of lineage U. The ancient genome (Troy) was used as the outgroup to determine the ancestral state. Synonymous, nonsynonymous, and intergenic sites were identified with SnpEff (98). The observed synonymous SFS contained an excess of singletons and high-frequency-derived variants. Both the observed SFS and the SFS predicted by the best-fitting model have an excess of singletons compared to the SFS predicted by the standard neutral model with no population size change.

Several human pathogens appear to have undergone recent population expansion (35–38). We wondered whether the uropathogenic lineage of *S. saprophyticus* might also have undergone a recent change in its effective size. The value for the genome-wide estimate of TD for lineage U was negative (−0.58), which is consistent with population expansion. We used the methods implemented in ∂a∂i (39) to identify the demographic model that best fit the observed synonymous SFS of lineage U (Fig. 7).

**FIG 8:**
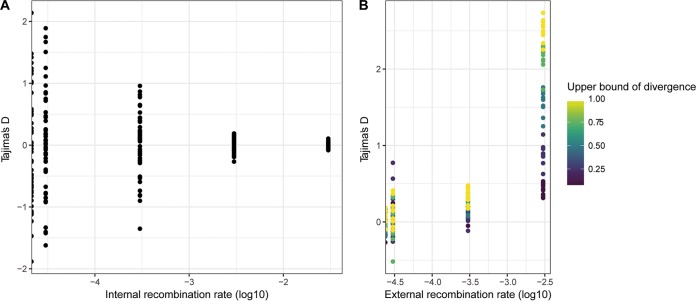
Effects of internal and external recombination on Tajima’s D values. Bacterial populations with a range of recombination rates were simulated with SimBac. (A) Tajima's D values from simulations of internal recombination rates ranging from 0 to 0.03 in the absence of external recombination. (B) Tajima’s D values from simulations performed with an internal recombination rate of 0.003 (r/m = 1) and external recombination rates ranging from 0 to 0.003. Points are filled according to the upper limit of diversity in external recombinant fragments.

The synonymous SFS showed an unexpected excess of high-frequency-derived alleles, which we hypothesized were the result of gene flow from populations with ancestral variants. Within-population recombination has been shown to have no effect on SFS-based methods of demographic inference in bacteria (40). However, external sources of recombination were not modeled in previous studies. We used SimBac (41) to simulate bacterial populations with a range of internal and external recombination rates. Similarly to previous studies, we found that within-population recombination had no effect on the value of Tajima’s D. However, we did find that recombinant tracts from external sources resulted in positive values of Tajima’s D (Fig. 8). Positive values of Tajima’s D are also associated with population bottlenecks and balancing selection.

**FIG 9:**
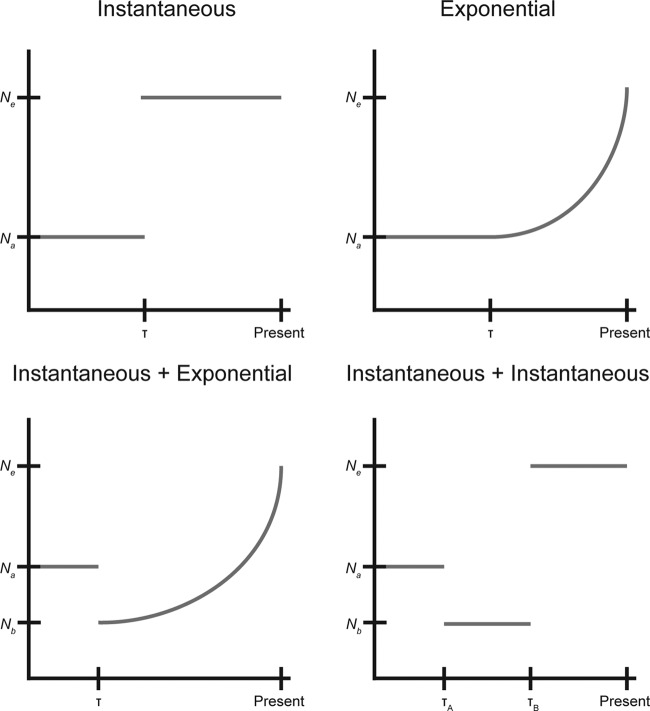
Cartoon of fitted demographic models. The observed synonymous SFS was fitted to 5 demographic models, including constant size, instantaneous population size change, exponential population size change, instantaneous population size change followed by exponential population size change, and two instantaneous population size changes. The parameters for the instantaneous and exponential models are the magnitude of the population size change (ν = *N*_*e*_/*N*_ancestral_) and the timing of the change (τ = number of generations/*N*_ancestral_). For the models with two population size changes, magnitudes are reported as ν_A_ = *N*_*b*_/*N*_ancestral_ and ν_b_ = *N*_*e*_/*N*_ancestral_.

We used fastGEAR (42) to identify recombinant tracts that originated outside lineage U, and these sites were removed from the analysis prior to demographic inference. We compared five demographic models (constant size, instantaneous population size change, exponential population size change, instantaneous population size change followed by exponential population size change, and two instantaneous population size changes; Fig. 9) and used bootstrapping to estimate the uncertainty of the parameters and to adjust the composite likelihoods using the Godambe Information Matrix implemented in ∂a∂i (43). We found significant evidence of expansion in all models (Table 2). The best-fitting model was an instantaneous contraction followed by an instantaneous expansion, in which the population underwent a tight bottleneck followed by a 15-fold expansion without recovering to its ancestral (anc) size (ν, *N*_*e*_/*N*_anc_; τ, number of generations/*N*_anc_; ν_A_, 2.9 × 10^−2^; ν_B_, 4.5 × 10^−1^; τ_A_, 1.2 × 10^−1^; τ_B_, 3.1 × 10^−3^).

**TABLE 2:** Results of demographic inference^a^

Model	Optimized parameters (SD)	Log likelihood	*P* value (comparison model)
Constant size		−562	
Instantaneous change	ν, 9.9 × 10^4^ (6.0 × 10^4^); τ, 4.4 × 10^−2^ (4.7 × 10^−3^)	−455	0.0 (constant size)
Exponential change	ν, 9.7 × 10^4^ (9.8 × 10^4^); τ, 4.1 × 10^−2^ (4.9 × 10^−3^)	−455	0.0 (constant size)
Instantaneous change followed by exponential change	ν_A_, 2.1 × 10^−2^ (2.9 × 10^−2^); ν_B_, 1.1 (3.9 × 10^−2^); τ, 1.3 × 10^−1^ (1.6 × 10^−1^)	−404	1.6 × 10^−4^ (exponential change)
Two instantaneous size changes	ν_A_, 2.9 × 10^−2^ (1.2 × 10^−2^); ν_B_, 4.5 × 10^−1^ (2.1 × 10^−1^); τ_A_, 1.2 × 10^−1^ (3.4 × 10^−2^); τ_B_, 3.1 × 10^−3^ (5.3 × 10^−3^)	−393	4.6 × 10^−7^ (instantaneous change)

^a^ ν = *N*_*e*_/*N*_ancestral_; τ = number of generations/*N*_ancestral_.

Recombination and positive selection are known to confound the inference of bacterial demography (40), so we used simulations to investigate their effects on our demographic inference performed for uropathogenic *S. saprophyticus*. We used SFS_CODE (44) to simulate positive selection (with a range of recombination rates) and to evaluate its effects on the accuracy of demographic inference with ∂a∂i. The method implemented in ∂a∂i relies on inference from the synonymous SFS, but it is possible for synonymous variation to be affected by selection, particularly at low rates of recombination (40, 45). Neutral simulations with gene conversion did not affect demographic inference. We did find that positive selection can affect the synonymous SFS, resulting in inference of population size changes. In simulations of positive selection in a population of constant size, we found the spurious inference to be a bottleneck rather than an expansion. This suggests that the observed synonymous SFS of lineage U has been affected both by positive selection and by demographic expansion.

## DISCUSSION

A central issue in the population biology of infectious diseases is how and why pathogenic traits emerge in microbes. Addressing this issue is important for understanding novel disease emergence and for identifying the genetic basis of virulence. Here we present evidence suggesting that a mutation in the *S. saprophyticus aas* gene, which binds host matrix proteins, is under positive selection and has enabled the emergence and spread of a human-pathogenic, UTI-associated lineage of this bacterium.

*S. saprophyticus* is familiar to medical microbiologists and clinicians as a common cause of UTI (46), which are associated with significant morbidity, economic costs, and severe complications (4). Despite its strong association with UTI in humans, *S. saprophyticus* can also be isolated from diverse environments, including livestock, food and food processing plants, and the environment (47, 48). Our previous research suggested that pathogenicity to humans is a derived trait in the species (7).

That pattern was replicated here, where phylogenetic analyses linked human UTI with two lineages of *S. saprophyticus* that are nested among isolates from diverse, nonhuman niches (i.e., the free-living and food- and animal-associated niches). The *aas* mutation arose in lineage U, which contains most of the UTI isolates. Two lineages are basal to lineage U; one is bovine associated, and the other contains an ancient bacterial sequence from a pregnancy-related infection in Late Byzantine Troy. The Troy bacterium has the ancestral, bovine-associated *aas* allele, and we have previously hypothesized (7) that this lineage could be associated with human infections in regions where humans have close contacts with animals—e.g., sharing living quarters with livestock, as they did at Troy during that time.

A second cluster of UTI isolates appears in clade E. One isolate has acquired the derived *aas* allele, which parallels our finding that two nonhuman isolates in lineage U acquired the ancestral variant; all of the recombination events that we observed at this locus reinforced the idea of an association between *aas*_2206A>C and human infection.

Several UTI isolates in clade E do not have the derived *aas* allele, and the clustering of UTI isolates suggests there may be a distinct adaptive path to virulence in this clade. Larger and more-comprehensive samples will be needed to investigate this hypothesis and to identify the factors shaping the separation of clades P and E.

The *aas* mutation has characteristics associated with a classical selective sweep driven by positive selection, namely, a regional reduction in diversity (21) and in Tajima’s D (22, 49). With the exception of the interesting allelic replacements noted above, there was also relatively little recombination at this locus, consistent with it being functionally important. To our knowledge, this is the first description of a single nucleotide sweep in a bacterium.

Depending on the strength of selection and the recombination rate, positive selection in bacteria has been observed to affect the entire genome, resulting in clonal replacements, or to affect only specific regions of the genome (50). For example, multiple clonal replacements have occurred in *Shigella sonnei* populations in Vietnam due to acquisition of resistance to antimicrobials and environmental stress (51). Recurrent clonal replacements have also been observed within single hosts during chronic infection of cystic fibrosis patients by *Pseudomonas aeruginosa* (52). Environmental bacterial populations can also be subject to clonal replacements; a metagenomic time course study of Trout Bog found evidence of clonal replacement occurring in natural bacterial populations but not gene- or region-specific sweeps (53). However, large regions of low diversity were also observed, suggesting that gene-specific selective sweeps had occurred prior to the start of the study. Shapiro et al. identified genomic loci that differentiated *Vibrio cyclitrophicus* isolates that were associated with distinct niches but that had limited diversity within niches; they concluded that differentiation of these populations had been enabled by recombination events that reinforced the association of alleles with the niche in which they were advantageous (54).

The *aas*_2206A>C mutation is among the genetic variants that differentiate bacteria associated with human-pathogenic niches from those associated with other niches (i.e., it is an *F*_*ST*_ outlier). SNPs associated with specific clinical phenotypes in the pathogen *Streptococcus pyogenes* were described recently (55), which is consistent with our finding that clinical phenotypes can represent distinct niche spaces preferentially occupied by subpopulations of bacteria. There is also precedent for a single nucleotide polymorphism to affect host tropism of bacteria (56).

In sexually reproducing organisms, haplotype-based statistics are frequently used to identify selective sweeps because positively selected alleles also increase the frequency of nearby linked loci faster than recombination can disrupt linkage, producing longer haplotypes for selected alleles (28, 57). We found that *aas*_2206A>C had a longer haplotype than the ancestral variant, but this difference was not extreme relative to the results seen with other regions of the genome (assessed with the *nS*_L_ statistic). Haplotype-based statistics have been found to perform poorly in analyses of purebred dogs, where linkage across the genome is high (58). Relatively low levels of recombination may also contribute to a lack of sensitivity when haplotype-based detection methods are applied to bacteria; linkage of sites is also likely to be disrupted in a less predictable way by bacterial gene conversion than by crossing over (29). Based on our findings, we conclude that screening for regional decreases in diversity and distortions of the SFS (i.e., sliding window analyses) and identification of genetic variants with extreme differences in frequency between niches can be useful in identifying candidate sites of positive selection in bacteria.

*S. saprophyticus* encodes a number of adhesins, including UafA, UafB, SdrI, and Aas. UafA and Aas are found in all isolates, suggesting that they play important roles in the diverse niches occupied by *S. saprophyticus*. Aas has autolytic, fibronectin binding, and hemagluttinating functions (9, 31, 32, 59). We identified a single, nonsynonymous polymorphism as a target of selection in the fibronectin binding repeats of *Aas*. This variant is predicted to affect the repeat’s structure, as proline has a more rigid structure than other amino acids. Adhesins are plausible candidates for adaptation to the uropathogenic niche, as they are known to be important virulence factors in pathogens causing urinary tract infections (60). Fibronectin binding proteins, including Aas, have been identified as virulence factors in *S. saprophyticus* and *Enterococcus faecalis* (32, 61, 62). Adhesion to the uroepithelium is essential for uropathogens to establish themselves in the bladder, where they are subject to strong shear stress (63): we hypothesize that *S. saprophyticus* strains with the derived *aas* variant are better able to colonize the human bladder.

Invasion of the human urinary tract may provide a fitness advantage by allowing relative enrichment of *S. saprophyticus* in a site with little competition from other bacterial species and by providing a mechanism of dispersal in the environment. In analyses of selection in *E. coli*, another bacterium occupying diverse niches, residues in the FimH adhesin were found to be subject to positive selection in uropathogenic strains (64–66). FimH binds mannose, providing protection from shear stress through a catch bond mechanism (67). Interestingly, the vascular adherence and resistance to shear stress of *Borrelia burgdorferi* were recently found to be enabled by interactions between a bacterial adhesin and host fibronectin that also use a catch bond mechanism (68). There are also precedents in *Staphylococcus aureus* for polymorphisms in bacterial fibronectin-binding adhesins to affect the strength of binding and for these polymorphisms to associate with specific clinical phenotypes (69).

Further experiments are needed to investigate the effects of variation in Aas on *S. saprophyticus* biology. In our preliminary investigations of binding using ELISAs of recombinant bacterial peptides, we did not detect differences between ancestral and derived alleles in binding of the R1a1b repeat with respect to fibronectin. The variant could still affect fibronectin binding by altering the conformation of the protein in a manner analogous to that seen with FimH in *E. coli* (66). It is also possible that variants in the peptide affect binding under specific conditions that we did not test. Another possibility is that the variant affects autolysis or other as-yet-undescribed functions of Aas. The roles of adhesins and other virulence factors in the colonization by *S. saprophyticus* of niches in livestock and the environment are also interesting topics for further study.

Our demographic analysis of the uropathogenic lineage of *S. saprophyticus* showed evidence of a population bottleneck and subsequent expansion. Bottlenecks and expansion of drug-resistant clones have previously been shown to affect the population structure of *Streptococcus agalactiae* (70), demonstrating the effects of positive selection on the demographic trajectories of bacterial subpopulations. However, previous work has also shown that selection—and recombination—can produce spurious results from demographic inference in bacteria (40, 71). We used an SFS-based method to reconstruct the demographic history of *S. saprophyticus*; the accuracy of demographic inference using these methods has been shown to be unaffected by within-population recombination (40), and this was confirmed in our analyses of simulated data. We found that recombination from external sources may result in an excess of intermediate frequency variants, which is also a signature of population bottlenecks, so we masked externally imported sites. However, the frequency of synonymous variants could still be affected by selection on linked nonsynonymous sites, including the selective sweep in *aas* that we have described. We performed simulations to address these potential confounders and to aid in the interpretation of our demographic inferences. Simulation of a single site under conditions of positive selection resulted in the inference of a bottleneck (*N*_*e*_/*N*_*a*_ = 0.01 to 0.42), indicating that, at the recombination rates that we simulated, diversity was lost from neutrally evolving sites due to their linkage to the site under selection. In inferences from our observed data, a bottleneck was followed by a 15-fold expansion, suggesting that lineage U has undergone both a selective sweep and demographic expansion.

Here we have described an adaptation of *S. saprophyticus* that may have enabled its expansion into a human-pathogenic niche. Mutation of a single nucleotide within the *aas* adhesin appears to have driven a selective sweep, and allele frequency differences at the locus are consistent with niche-specific adaptation. Lateral gene transfer events in *aas* reinforced the association of the positively selected allele with human infection. These results provide new insights into the emergence of virulence in bacteria and outline an approach for discovering the molecular basis of adaptation to the human-pathogenic niche.

## MATERIALS AND METHODS

### DNA extraction.

After overnight growth in tryptic soy broth (TSB) at 37°C in a shaking incubator, cultures were pelleted and resuspended in 140 μl of Tris-EDTA (TE) buffer. Cells were incubated overnight with 50 units of mutanolysin. We used a MasterPure Gram-positive DNA purification kit (EpiCentre) for DNA extraction. For DNA precipitation, we used 1 ml 70% ethanol and centrifugation at 4°C for 10 min. We additionally used a SpeedVac for 10 min to ensure that pellets were dry before resuspending the pellet in 50 µl of water.

### Library preparation and sequencing.

For SSC01, SSC02, and SSC03, library preparation was performed using a modified Nextera protocol as described by Baym et al. (72) with a reconditioning PCR with fresh primers and polymerase for an additional 5 PCR cycles to minimize chimeras and two-step bead-based size selection with a target fragment size of 650 bp and sequencing on an Illumina HiSeq 2500 sequencer (paired end; 150 bp). For 43, SSC04, SCC05, and SSMast, DNA was submitted to the University of Wisconsin—Madison Biotechnology Center for library preparation and was prepared using a TruSeq Nano DNA LT Library Prep kit (Illumina Inc., San Diego, CA) with minor modifications. A maximum of 200 ng of each sample was sheared using a Covaris M220 Ultrasonicator (Covaris Inc., Woburn, MA). Sheared samples were size selected for an average insertion size of 550 bp using Spri bead-based size exclusion. The quality and quantity of the finished libraries were assessed using an Agilent DNA High Sensitivity chip (Agilent Technologies, Santa Clara, CA) and an Qubit dsDNA HS assay kit, respectively. Libraries were standardized to 2 μM. Paired-end, 150-bp sequencing was performed using v2 SBS chemistry on an Illumina MiSeq sequencer. Images were analyzed using the Illumina Pipeline, version 1.8.2.

### Reference guided mapping.

We mapped reads to ATCC 15305 via the use of a pipeline (available at https://github.com/pepperell-lab/RGAPepPipe). Briefly, read quality was assessed and reads were trimmed with TrimGalore! v 0.4.0 (http://www.bioinformatics.babraham.ac.uk/projects/trim_galore), which runs both FastQC (http://www.bioinformatics.babraham.ac.uk/projects/fastqc) and cutadapt. Reads were mapped using BWA-MEM v 0.7.12 (73) and sorted using Samtools v 1.2 (74). We used Picard v 1.138 (http://broadinstitute.github.io/picard/) to add read group information and removed duplicates. Reads were locally realigned using GATK v 2.8.1 (75). We identified variants using Pilon v 1.16 (76) (minimum read depth, 10; minimum mapping quality, 40; minimum base quality, 20).

### Assembly.

We used the iMetAMOS pipeline for *de novo* assembly (77). We chose to compare assemblies from SPAdes (78), MaSurCA (79), and Velvet (80). KmerGenie (81) was used to select kmer sizes for assembly. iMetAMOS uses FastQC, QUAST (82), REAPR (83), LAP (84), ALE (85), FreeBayes (86), and CGAL (87) to evaluate the quality of reads and assemblies. We also used Kraken (88) to detect potential contamination in sequence data. For all newly assembled isolates (43, SSC01-05, SSMast), the SPAdes assembly was the highest quality. Assembly statistics are reported in Table S2 in the supplemental material.

10.1128/mSphere.00511-17.2 TABLE S2  Assembly statistics for *S. saprophyticus* genomes. Download TABLE S2, TXT file, 0.01 MB. Copyright © 2017 Mortimer et al. 2017 Mortimer et al. This content is distributed under the terms of the Creative Commons Attribution 4.0 International license.

### Annotation and gene content analyses.

We annotated the *de novo* assemblies using Prokka v 1.11 (89) and used Roary (90) to identify orthologous genes in the core and accessory genomes. To look for associations between accessory gene content and human association, we used Scoary (19). For the analysis, we used human association as the trait of interest, and we adjusted the *P* value for multiple comparisons using the Bonferroni method.

### Alignment.

When short-read data for reference guided mapping were unavailable, whole-genome alignment of genomes to ATCC 15305 was performed using Mugsy v 2.3 (91). Repetitive regions in the reference genome that were greater than 100 bp in size were identified using nucmer, and these regions were masked in the alignment used in downstream analyses.

### Maximum likelihood phylogenetic analysis.

Maximum likelihood phylogenetic trees were inferred using RAxML 8.0.6 (92). We used the GTRGAMMA substitution model and performed bootstrapping using the autoMR convergence criteria. Tree visualizations were created in ggtree (93).

### Population genetics statistics.

To calculate π and Tajima’s D, we used EggLib v 2.1.10 (94), a Python package for population genetic analyses. A script to perform the sliding window analysis is available at https://github.com/tatumdmortimer/popgen-stats/blob/master/slidingWindowStats.py. We used vcflib (https://github.com/vcflib/vcflib) to calculate *F*_*ST*_ and EHH and selscan v 1.1.0b (95) to calculate *nS*_L_.

### Recombination analyses.

To identify recombinant regions in the *S. saprophyticus* alignment, we used Gubbins v 2.1.0 (20). fastGEAR (42) was used with the recommended input specifications to identify recombination events between major lineages of *S. saprophyticus*. We used Circos (96) for visualization of recombinant tracts.

### Site frequency spectrum.

We used SNP-sites v 2.0.3 (97) to convert the alignment of *S. saprophyticus* isolates to a multisample variant call format (VCF). SnpEff v 4.1j (98) was used to annotate variants in this VCF as synonymous, nonsynonymous, or intergenic. Using the Troy genome as an outgroup, we calculated an unfolded site frequency spectrum (SFS) for lineage U for each category of sites. To reduce the impact of lateral gene transfer on the SFS, we removed sites where the origin was outside lineage U based on results of fastGEAR analysis.

### Ancestral reconstruction of aas_2206A>C.

We used TreeTime (99) to reconstruct the evolutionary history of the variant in *aas* using the maximum likelihood phylogeny inferred using RAxML.

### Demography.

We performed demographic inference with the synonymous SFS using ∂a∂i (39). The models tested were the standard neutral model and the expansion and exponential-growth models. The parameters *ν* (*N*_*e*_/*N*_*a*_) and *τ* (time scaled by 2) were optimized for both the expansion and exponential-growth models. The statistical significance of results of comparisons of data from the expansion and exponential-growth models to data from the standard neutral model was evaluated using a likelihood ratio test. The scripts used to perform this analysis are available at https://github.com/tatumdmortimer/popgen-stats.

### Simulations.

Simulations were performed in SimBac (41) to evaluate the effect of external recombination on the SFS. Populations were simulated with sample size and θ equivalent to those seen with our sample of lineage U (*n* = 44; θ = 0.003). The length of internal recombinant tracts was 6,500 bp (median of Gubbins output), and the length of external recombination events was 3,000 bp (median of fastGEAR output). Internal recombination was simulated at rates ranging from 0 to 0.03 (r/m = 10). External recombination was simulated at rates ranging from 0 to 0.003. The lower bound of difference for external recombination was 0, and the upper bound was simulated at values ranging from 0.25 to 1.0. Simulations were performed in SFS_CODE (release date, 10 September 2015) (44) to evaluate the power of ∂a∂i to accurately estimate demographic parameters in the presence of gene conversion and selection. We simulated a locus of length of 100 kb with theta of 0.003, a gene conversion tract length of 1,345 bp, and a range of recombination/mutation ratios (0.0002 to 2.0). In addition to neutral simulations with gene conversion, we also performed simulations with a single site under conditions of selection (γ = 10 to 1,000) with the same parameters as those used for the neutral simulations.

### Expression of R1ab.

Human-associated and ancestral strain R1ab bacteria were cloned into expression vector pET-ELMER (102), transformed into BL21(DE3) cells (EMD, Gibbstown, NJ) for expression, and induced with 1 mM IPTG (isopropyl-β-d-thiogalactopyranoside). Bacteria were lysed in lysis buffer consisting of 100 mM NaH_2_PO_4_, 10 mM Tris, 8 M urea, 1 mM β-mercaptoethanol, and 5 mM imidazole (pH 8.0). The cleared lysate was incubated overnight with nickel-nitrilotriacetic acid agarose (Qiagen), washed, and eluted in lysis buffer (pH 7.0)–300 mM imidazole.

### ELISA.

Antigen was diluted to 10 µg/ml in Tris-buffered saline (TBS; 10 mM Tris, 150 mM NaCl, pH 7.4) and used to coat 96-well microtiter plates (Costar 3590 high binding; Corning Inc., Corning, NY) with 50 µl per well for 16 h at 4°C. The plates were blocked with 1% bovine serum albumin (BSA)–TBS–0.05% Tween 20 (TBST) for 1 h. After washing with TBST was performed three times, purified plasma fibronectin (100) or platelet-derived thrombospondin-1 (101) diluted to 10, 3, 1, or 0.3 µg/ml in TBST–0.1% BSA was added to the plates and the reaction mixture was incubated for 2 h. Plates were washed four times with TBST. Rabbit anti-fibronectin and rabbit anti-thrombospondin antibodies diluted in TBST–0.1% BSA were added to the appropriate wells, and the reaction mixtures were incubated for 1 h. Plates were washed four times with TBST. Peroxidase-conjugated secondary antibody was incubated with the plates for 1 h. Plates were washed four times with TBST, and 50 µl of SureBlue TMB peroxidase substrate (KLP) per well was added to each well. Color development was monitored for 10 to 30 min, and 50 µl of TMB stop solution (KLP) was added, followed by measurement of absorbance at 450 nm.

### Accession number(s).

The new accession numbers for the data from the *S. saprophyticus* isolates are provided in Table S1 in the supplemental material.
